# Inflammation of actinic keratosis with combination of alkylating and taxane agents: a case report

**DOI:** 10.4076/1757-1626-2-6946

**Published:** 2009-07-02

**Authors:** Fadi Makdsi, Roger DeVersa

**Affiliations:** Department of Medicine, University of Tennessee, College of MedicineChattanoogaUSA

## Abstract

**Introduction:**

Carboplatin and docetaxel are chemotherapy agents that are used to treat some malignancies. This combination has not been documented to date to cause an inflammation of a preexisting actinic keratosis. One previous report has described inflammation of AK lesions after the use of docetaxel alone.

**Case presentation:**

We report a case of a 54-year-old male with a history of untreated actinic keratosis and lung cancer. He developed a rash on the sun exposed areas of skin after he received carboplatin and docetaxel. Topical high-potency corticosteroid administration resulted in diminished rash despite the ongoing chemotherapy regimen.

**Conclusion:**

We present this case to emphasis this potential reaction in predisposed individuals after using the combination of carboplatin and docetaxel, and to help physicians making the appropriate decision whether to continue the chemotherapy or discontinued it.

## Introduction

Actinic keratosis (AK) is a common skin lesion that occurs in the elderly on sunlight exposed skin surfaces. Inflammation of actinic keratosis secondary to chemotherapy agents is a known phenomenon, most commonly occurring with fluorouracil. To our knowledge, there has been no report about the association between actinic keratosis inflammation with the combination of carboplatin and docetaxel.

## Case presentation

**Figure 1.**
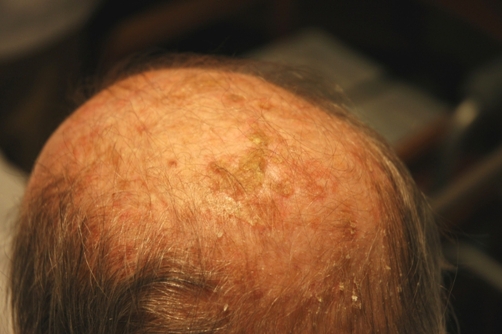
Patient scalp shows yellow, and scaly lesions consistent with AK.

**Figure 2.**
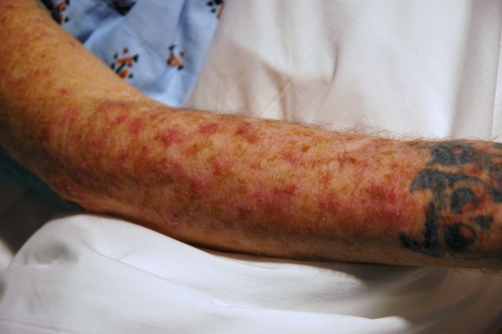
Patient right arm shows numerous scaly, and erythematous nodules consistent with inflamed AK.

A 54-year-old Caucasian male presented with a one day history of rash. The rash was on his upper trunk and extensor surfaces of his arms. It was accompanied by a burning sensation, but was otherwise non-tender to palpation and non-pruritic. He had a history of COPD, Hepatitis C, and previously unrecognized actinic keratosis. He also had newly diagnosed adenocarcinoma of the lung treated six days prior to presentation with a second cycle of chemotherapy. The combination of carboplatin (798 mg) and docetaxel (130 mg) was used for his regimen. Physical exam revealed a chronically ill-appearing man with no acute distress and intact oral mucosa. Skin examination was notable for excessive dryness and multiple skin lesions. The scalp had yellow, sharply bordered, scaly lesions (Figure 1). The extensor surfaces of both arms (Figure 2), as well as the back of the neck, had numerous non-tender nodules. The lesions were adherent, scaly, erythematous, ranging from 5-10 mm in size, and circular to ovoid in shape. This rash was consistent with an inflamed actinic keratosis eruption secondary to his recent chemotherapy. The rash improved with the treatment of desonide cream 0.05%.

## Discussion

Actinic keratosis is a common skin lesion that occurs in the elderly on sunlight exposed skin surfaces. These lesions are confined to the epidermis, but when they extend deeply into the dermis, they called squamous cell carcinoma. Numerous methods of AK treatment have been described depending on the extent of the disease. They can include cryotherapy, surgical removal, or topical drugs like imiquimod and 5-fluorouracil (5-FU). 5-FU is successful as a topical agent because it diffuses inside rapidly replicating cells and kills them with minimal effect on the normal cells. Toxic reaction and inflammation of the lesions is known to occur during the early stages of treatment. The inflammation can start within 1-2 weeks of beginning treatment and it can include different phases [1]. Different systemic chemotherapy agents and combination protocols are also known to produce an inflammation of the actinic keratosis via unclear mechanism. Radiation recall reaction and susceptible cell with abnormal DNA have been suggested to explain AK inflammation [2]. The most commonly described systemic chemotherapy agent that can cause a flair of actinic keratosis is 5-Fu [2]. Other cited agents include dactinomycin-dacarbazine-vincristine combination, doxorubicin-cytarabine-thioguanine combination, sorafenib-tipifarnib combination, capecitabine, doxorubicin, erlotinib, deoxycoformycin, fludarabine, and cisplatin [2-6]. Docetaxel, one of the taxane agents, has been described in one report to cause inflammation of actinic keratosis in two patients [7]. No association between AK inflammation and the alkylating agent carboplatin has been found in the literature. To our knowledge, this is the first case report describing an association between actinic keratosis inflammation and the combination of carboplatin and docetaxel.

Our patient suffered from actinic keratosis inflammation after his second chemotherapeutic treatment for lung cancer using combination therapy. Certainly, it is known that docetaxel alone may induce this response. We cannot discern if the combination of these agents lead to a more robust flair than if he had received solely docetaxel. It should be investigated whether the sole use of carboplatin also has a similar effect on AK.

## Conclusion

Chemotherapy agents have been widely used to treat malignancies, and many agents continue to be discovered and added. Because of the time delay that occurs between introducing new drugs and the documentation of their new reactions, physicians, particularly oncologists and dermatologists, should be aware of the potential cutaneous adverse effects of these agents. Inflammation of preexisting and subclinical actinic keratosis is an effect of particular interest. Screening for actinic keratosis prior to initiating chemotherapy may be beneficial, particularly if agents that are known to cause inflammation of AK are being considered. This may provide an opportunity for anticipatory guidance regarding a possible flair after induction, and may prevent misdiagnosis of a drug reaction with unnecessary skin biopsy or termination of potentially life saving treatment.

## Statement of proprietary interest

The authors hereby state that there are no commercial or proprietary interests of any kind of any drug, device, or equipment mentioned in this study. Neither author has any financial interest of this study.

## Abbreviations

AK: actinic keratosis
5-FU: 5-fluorouracil

## References

[bib-001] Habif TP, Habif TP (2004). Clinical Dermatology: A color guide to diagnosis and therapy.

[bib-002] Susser WS, Whitaker-Worth DL, Grant-Kels JM (1999). Mucocutaneous reactions to chemotherapy. J Am Acad Dermatol.

[bib-003] Peramiquel L, Dalmau J, Puig L, Roé E, Fernández-Figueras MT, Alomar A (2006). Inflammation of actinic keratoses and acral erythrodysesthesia during capecitabine treatment. J Am Acad Dermatol.

[bib-004] Hermanns JF, Piérard GE, Quatresooz P (2007). Erlotinib-responsive actinic keratoses. Oncol Rep.

[bib-005] Hong DS, Reddy SB, Prieto VG, Wright JJ, Tannir NM, Cohen PR, Diwan AH, Evans HL, Kurzrock R (2008). Multiple squamous cell carcinomas of the skin after therapy with sorafenib combined with tipifarnib. Arch Dermatol.

[bib-006] Remlinger KA (2003). Cutaneous reaction to chemotherapy drugs: the art of consultation. Arch Dermatol.

[bib-007] Zimmerman GC, Keeling JH, Burris HA, Cook G, Irvin R, Kuhn J, McCollough ML, Von Hoff DD (1995). Acute cutaneous reactions to docetaxel, a new chemotherapeutic agent. Arch Dermatol.

